# Left Atrioventricular Coupling Index: A Comprehensive Review of the Literature

**DOI:** 10.3390/life16050722

**Published:** 2026-04-24

**Authors:** Nikolaos Tsiamis, Dimitrios Afendoulis, Christos Tountas, Christo Kole, Flora Tsakirian, Fotios Toulgaridis, Ioannis Kachrimanidis, Anastasios Apostolos, Asimina Romiou, Nikolaos Ktenopoulos, Maria Drakopoulou, Anastasia Kitsiou, Konstantina Aggeli, Konstantinos Tsioufis, Konstantinos Toutouzas

**Affiliations:** 1 1st Department of Cardiology, National and Kapodistrian University of Athens (NKUA), ‘Hippokration’ General Hospital of Athens, 11527 Athens, Greece; loratsakirianmed@gmail.com (F.T.); iskachrimanidis@gmail.com (I.K.); nikosktenop@gmail.com (N.K.); ktoutouz@gmail.com (K.T.); 2 Cardiology Department, Sismanogleio General Hospital, 15126 Athens, Greecechristo.kole@gmail.com (C.K.); fotistoulgaridis@gmail.com (F.T.);; 3 Department of Cardiology, Harefield Hospital, Royal Brompton and Harefield Hospitals, Guy’s and St Thomas’ NHS Foundation Trust, London UB9 6JH, UK; anastasisapostolos@gmail.com

**Keywords:** left atrioventricular coupling index (LACI), atrioventricular uncoupling, cardiac imaging, chamber interaction, left atrial volume, heart failure, prognostic risk stratification

## Abstract

Traditional cardiovascular assessment has historically focused on the isolated evaluation of either atrial or ventricular structure and function. However, the left atrioventricular coupling index (LACI) represents a paradigm shift by moving beyond single-chamber metrics to quantify the dynamic interaction between the left atrium and left ventricle. Defined as the ratio of left atrial end-diastolic volume to left ventricular end-diastolic volume, LACI integrates structural and functional aspects of cardiac performance. This comprehensive review examines the physiological basis of how the left atrium and ventricle operate as an integrated hemodynamic unit. We detail current measurement methodologies, including two- and three-dimensional echocardiography, cardiac computed tomography, and cardiac magnetic resonance imaging, which serves as the reference standard. Furthermore, the review explores the pathophysiological mechanisms driving atrioventricular uncoupling, specifically mechanical dysfunction, electromechanical desynchrony, and hemodynamic alterations. Extensive clinical evidence demonstrates LACI’s robust independent prognostic value across diverse cardiovascular conditions, such as heart failure, myocardial infarction, cardiomyopathies, and atrial fibrillation. Observational data suggest that LACI provides a promising prognostic value beyond established risk assessment tools by combining the assessment of both chambers’ interdependence. Finally, we outline future directions for clinical translation, highlighting the necessity for standardized measurement protocols, the integration of artificial intelligence, and the potential of LACI as a target for personalized therapeutic strategies.

## 1. Introduction

Traditional cardiovascular assessment has historically focused on the isolated evaluation of either atrial or ventricular structure and function. However, this single-chamber approach presents significant physiological and clinical limitations. Cardiac function is fundamentally dependent on coordinated chamber interaction rather than isolated performance. Traditional metrics, such as evaluating left atrial volume index or left ventricular ejection fraction independently, inherently fail to capture the dynamic, real-time interaction between these chambers. This isolated perspective can mask early pathophysiological changes and falls short of aligning with the contemporary understanding of cardiovascular disease, particularly heart failure, where the disruption of chamber interaction plays a central role in disease progression. Because the physiological relationship between the left atrium and ventricle is so intimately linked, assessing their coupling better reflects left atrioventricular dysfunction and provides superior prognostic information compared to traditional single-chamber metrics [1].

The left atrioventricular coupling index (LACI) represents a paradigm shift in cardiovascular assessment, moving beyond traditional isolated chamber evaluation to quantify the dynamic interaction between the left atrium and left ventricle [1]. Defined as the ratio of left atrial end-diastolic volume to left ventricular end-diastolic volume during the mitral valve closure phase: *LACI* = $\frac{Left\;Atrial\;End\;Diastolic\;Volume}{Left\;Ventricular\;End\;Diastolic\;Volume}\;$ [2]. Physiologically, this index provides a comprehensive measure of atrioventricular function that integrates both structural and functional aspects of cardiac performance. It encapsulates the complex physiological relationship between the left atrium and ventricle, offering vital insights into overall cardiac hemodynamics that neither atrial nor ventricular parameters can provide on their own [3].

The conceptual foundation of LACI rests on the understanding that cardiac function is fundamentally dependent on coordinated chamber interaction rather than isolated performance [4]. Atrioventricular (AV) coupling refers to the functional synchronization of atrial and ventricular activity during both systole and diastole, ensuring efficient filling and ejection throughout the cardiac cycle. The atria and ventricles are intimately linked; their interaction extends beyond simple reservoir function to a complex, time-dependent exchange of volume and pressure. During ventricular systole, atrial filling is facilitated by the downward displacement of the atrioventricular (mitral/tricuspid) annulus, which defines the atrial reservoir phase. In early diastole, the atria act as a passive conduit, transferring blood to the ventricles—a process largely determined by ventricular relaxation and compliance. Finally, in late diastole, active atrial contraction augments ventricular preload, enhancing both stroke volume and contractility. In normal sinus rhythm, this coordinated atrial systole contributes approximately 20% to 30% of the ventricular stroke volume, underscoring the critical importance of AV synchrony for optimal hemodynamics. Therefore, this interplay relies not only on atrial contractility and compliance but also on ventricular compliance and filling pressures, establishing a vital, bidirectional relationship.

From a hemodynamic perspective, diastole begins with the opening of the mitral valve, allowing blood to flow from the left atrium (LA) to the left ventricle (LV). At this point, rotational flow within the LA dissipates, giving way to the formation of vortex flow in the LV. This vortex flow—which is initially stronger than the preceding rotational flow in the LA during early diastole—generates kinetic energy that facilitates LV filling by aiding its diastolic volume expansion. During late diastole, this vortex flow organizes and optimizes blood flow from the LA toward the LV outflow tract, actively aiding in the stretching of cardiomyocytes to enhance subsequent ventricular contraction. These complex fluid interactions are critical for maintaining optimal preload adaptation and ensuring effective ventricular performance. Together, this physiological and hemodynamic interplay between the LA and LV highlights the central, indispensable role of LA-LV coupling in overall cardiac function. This integrative approach aligns with contemporary understanding of heart failure pathophysiology, where disruption of chamber interaction plays a central role in disease progression [5].

## 2. Objectives/Methods

**Figure 1:**
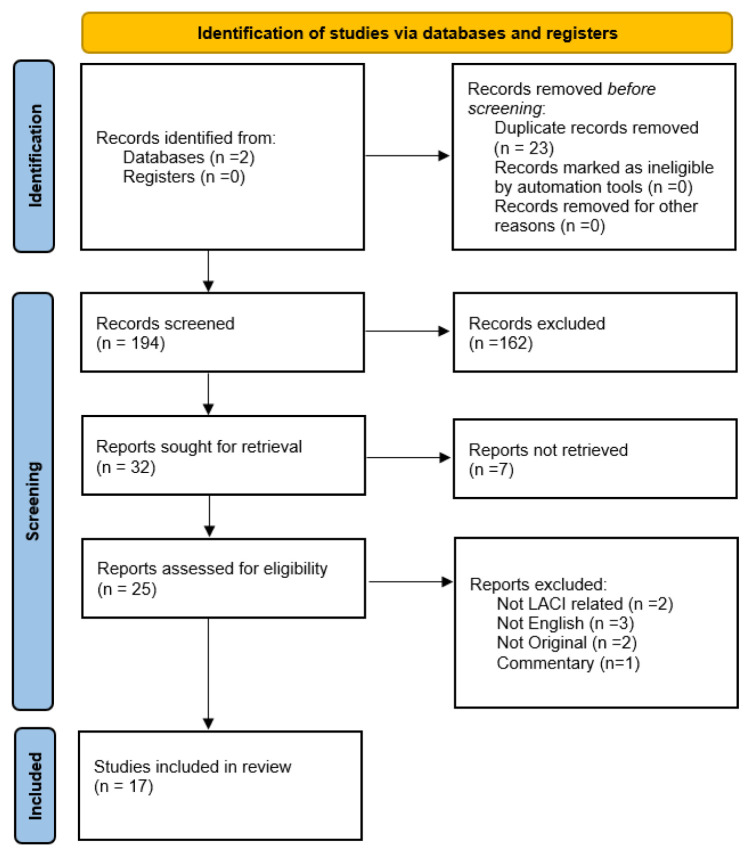
PRISMA flowchart used for our review.

The aim of our review was to conduct detailed literature research regarding LACI, given the fact that this is a relatively new index used by some imaging centers for systematic evaluation of cardiac function and summarizing all the available evidence as well as its use in clinical practice and its potential role for screening the population for early ventricular dysfunction. Research was done across two main platforms (PubMed and Google Scholar), from two members of our team, using terms “Left Atrioventricular Coupling Index”, from January 2010 until February 2026 following the PRISMA guidelines (Figure 1). Two platforms were used for our review given that the literature regarding LACI is limited and information was found across these two platforms. All the abstracts, full texts and references sections of the available articles were screened for relevance and potential sources of more information.

### Eligibility Criteria, Screening and Data Extraction

Studies were eligible for inclusion if they focused on Left Atrioventricular Coupling Index, and applications on imaging modalities, or clinical settings. Exclusion criteria included duplicates, non-English articles, abstracts with no available main text, non-human studies and irrelevant articles. Moreover, full texts of potentially eligible studies were assessed by the same reviewers. Key data were extracted by the reviewers, focusing on study and population characteristics, and application of LACI in clinical practice or comparison with established imaging modalities.

## 3. Results and Discussion

Out of the 194 articles initially found during our research, 25 articles were screened for relevance and 17 articles meeting our inclusion criteria were included in our review.

LACI emerged from large population-based studies demonstrating that the ratio of atrial to ventricular volumes provides incremental prognostic value beyond traditional risk factors [6,7]. The Multi-Ethnic Study of Atherosclerosis (MESA) was instrumental in establishing LACI as an independent predictor of incident heart failure, with both baseline values and longitudinal changes showing strong associations with adverse outcomes [8]. Subsequent validation across diverse clinical populations has confirmed that LACI captures unique pathophysiological information not reflected by isolated chamber measurements [9].

### 3.1. Measurement Techniques and Methodologies

#### 3.1.1. Echocardiographic Assessment (2D, 3D, AI-Enhanced)

Two-dimensional echocardiography represents the most accessible modality for LACI assessment, leveraging standard transthoracic imaging protocols without requiring additional sequences [10,11,12,13]. LACI is calculated from apical two- and four-chamber views using the biplane Simpson’s method to quantify left atrial and ventricular end-diastolic volumes [9,10]. The simplicity of this approach facilitates widespread clinical implementation, with measurements typically under two minutes [14]. However, two-dimensional echocardiography has inherent limitations related to geometric assumptions, particularly in patients with atrial or ventricular remodeling that distorts chamber geometry [15]. Despite these constraints, studies demonstrate good feasibility and reproducibility, with LACI measurable in over 95% of patients with adequate image quality [14].

Three-dimensional (3D) echocardiography overcomes many limitations of two-dimensional imaging by providing direct volumetric quantification without geometric assumptions [15]. 3D echocardiography eliminates the need for geometric modeling, thereby reducing inter-observer variability and shortening analysis times. Real-time three-dimensional acquisition enables comprehensive assessment of chamber volumes and coupling indices with superior accuracy compared to two-dimensional methods [15]. Studies employing three-dimensional echocardiography report excellent correlation with cardiac magnetic resonance, the gold standard for volumetric assessment [16]. The combined atrioventricular coupling index, incorporating both left and right heart coupling, can be readily measured using three-dimensional techniques, providing a more comprehensive assessment of global cardiac function [15] (Table 1).

#### 3.1.2. Cardiac Magnetic Resonance Imaging

Cardiac magnetic resonance (CMR) provides the reference standard for LACI assessment, offering superior spatial resolution, tissue characterization, and reproducibility compared to echocardiographic techniques [1]. Standard cine imaging protocols using balanced steady-state free precession sequences enable precise quantification of left atrial and ventricular volumes throughout the cardiac cycle [12]. The LACI is derived by calculating the ratio of the LA volume to the LV end-diastolic volume, with both parameters meticulously measured via CMR, with excellent inter- and intra-observer reproducibility [5]. The LA volume is determined using the area-length method from 2- and 4-chamber views, with the biplane calculation performed using the following formula: LA volume = (0.848 × Area^2ch^ × Area^4ch^)/[(LA Length^2ch^ + Length^4ch^)]/2). The ability to obtain accurate volumetric data independent of chamber geometry makes CMR particularly valuable in patients with structural heart disease where echocardiographic assessment may be limited [13].

Beyond volumetric assessment, CMR provides unique insights into the mechanisms underlying atrioventricular uncoupling through advanced tissue characterization [17]. T1 mapping and extracellular volume quantification detect diffuse myocardial fibrosis in both atrial and ventricular myocardium, which correlates with impaired coupling and adverse outcomes [16,18]. Late gadolinium enhancement imaging identifies focal fibrosis and scar burden, important determinants of atrial and ventricular remodeling [12]. Feature-tracking strain analysis derived from standard cine images enables comprehensive assessment of chamber deformation, providing complementary information to volumetric LACI [8]. Exercise stress CMR represents an emerging application, with studies demonstrating that LACI measured during physiological stress improves diagnostic accuracy for heart failure with preserved ejection fraction [17,19]. The correlation between LACI and pulmonary capillary wedge pressure during exercise stress (r = 0.55, *p* < 0.001) highlights its value as a noninvasive surrogate for invasive hemodynamic assessment [17] (Table 1).

#### 3.1.3. Cardiac Computed Tomography

Cardiac computed tomography (CT) has emerged as an alternative modality for LACI assessment, particularly attractive given its widespread availability and integration into coronary artery disease evaluation protocols [20]. Standard coronary CT angiography acquisitions provide adequate temporal and spatial resolution for volumetric quantification of both atrial and ventricular chambers without requiring dedicated protocols [13]. Using electrocardiographic gating, the cardiac cycle is specifically analyzed to identify end-diastolic frames corresponding to the closure of the mitral valve. The Simpson’s method is applied to two- and four-chamber views to calculate the volumes of the LA and LV at end-diastole. Studies demonstrate good feasibility of LACI measurement from cardiac CT, with strong correlation to CMR-derived values [20]. This is particularly relevant for patients undergoing CT for coronary assessment, enabling simultaneous evaluation of anatomic disease burden and functional coupling indices [21]. Cardiac CT provides distinct benefits in specific clinical scenarios due to its quick scan times and excellent spatial detail. Nevertheless, its application is hindered by significant drawbacks, primarily the exposure to ionizing radiation and the potential hazards of contrast agents.

The prognostic value of CT-derived LACI has been validated in patients without known cardiovascular disease referred for coronary CT angiography [20]. In a cohort of 1444 patients followed for nearly 7 years, LACI independently predicted cardiovascular death and all-cause mortality with incremental value over traditional risk factors and coronary findings [20]. A LACI threshold of 25% showed optimal discrimination for cardiovascular death, with significant improvement in model performance when added to conventional risk assessment [20]. The ability to derive LACI from routine cardiac CT acquisitions without additional radiation or contrast exposure enhances its clinical utility and cost-effectiveness [21] (Table 1).

### 3.2. Pathophysiological Pathways of LACI

#### 3.2.1. Mechanical Dysfunction

Atrial dilatation represents one of the primary mechanical mechanisms driving atrioventricular uncoupling [1]. Progressive left atrial enlargement occurs in response to chronic pressure and volume overload, initially serving as a compensatory mechanism to maintain adequate ventricular filling [6]. However, persistent atrial stretch leads to structural remodeling characterized by myocyte hypertrophy, interstitial fibrosis, and extracellular matrix deposition [10]. Studies using CMR tissue characterization demonstrate that increased left atrial extracellular volume correlates strongly with elevated LACI and adverse outcomes [16].

**Figure 2:**
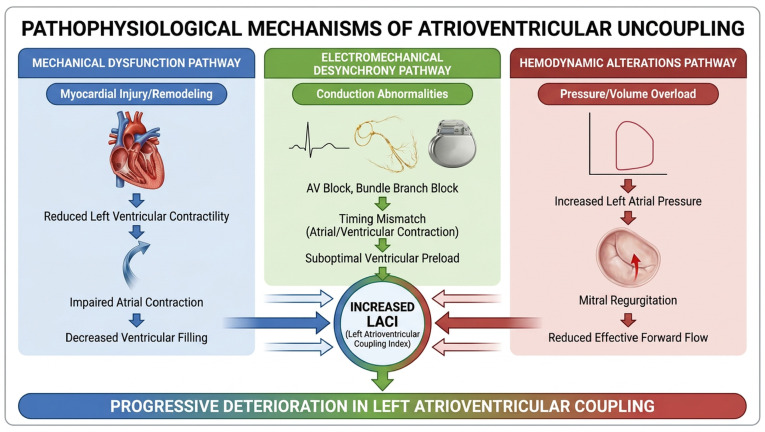
Pathophysiological Mechanisms of Atrioventricular Uncoupling. The figure illustrates the three major pathways leading to atrioventricular uncoupling and increased LACI: mechanical dysfunction, electromechanical desynchrony, and hemodynamic alterations. These mechanisms interact to produce progressive deterioration in left atrioventricular coupling.

Atrial fibrosis emerges as a key substrate for atrioventricular uncoupling, detectable through late gadolinium enhancement on CMR or electroanatomic voltage mapping [10]. Fibrotic infiltration disrupts normal myocardial architecture, reducing atrial contractility and compliance while promoting electrical instability [11]. The extent and distribution of atrial fibrosis correlate with LACI values and predict progression to atrial fibrillation [22]. Ventricular diastolic stiffness represents the other major mechanical contributor to uncoupling, creating increased afterload for atrial emptying and reducing the pressure gradient driving ventricular filling [17] (Figure 2).

#### 3.2.2. Electromechanical Dysynchrony

Electromechanical desynchrony disrupts the normal temporal coordination between atrial and ventricular contraction, impairing optimal filling dynamics [1]. Atrioventricular block and prolonged PR interval reduce ventricular filling time and may cause atrial contraction to occur against a closed mitral valve, eliminating the booster pump contribution [23]. This diastolic mitral regurgitation, visible on echocardiography as presystolic flow reversal, represents a hemodynamic consequence of AV dyssynchrony that directly impairs coupling [24]. Studies in patients requiring pacemakers demonstrate that right ventricular pacing-induced dyssynchrony adversely affects LACI compared to physiological conduction [23].

Atrial conduction delay, quantified as total atrial conduction time or P-wave duration, represents another manifestation of electromechanical uncoupling [25]. Prolonged intra-atrial and inter-atrial conduction creates temporal dispersion of atrial activation and contraction, reducing the efficiency of coordinated chamber emptying [11]. Ventricular dyssynchrony, particularly in the setting of left bundle branch block or pacing-induced activation abnormalities, further impairs coupling by reducing the suction effect of early diastolic ventricular relaxation [23]. Paradoxically, some studies report worsening LACI following cardiac resynchronization therapy despite clinical improvement, suggesting complex relationships between electrical optimization and chamber coupling [24] (Figure 1).

#### 3.2.3. Relationship with Diastolic Dysfunction

LACI demonstrates strong associations with left ventricular diastolic dysfunction across multiple diagnostic frameworks [26]. In a cohort of 1158 heart failure patients, LACI progressively increased across diastolic dysfunction grades, with grade 3 dysfunction showing markedly elevated values compared to grade 1 [26]. A LACI threshold of 0.26 identified moderate-to-severe diastolic dysfunction with an area under the curve of 0.75, demonstrating good discriminative ability [26]. The relationship between LACI and diastolic dysfunction reflects shared pathophysiological mechanisms, as impaired ventricular relaxation and increased filling pressures directly impact atrial function and remodeling [17]. Studies using exercise stress demonstrate that LACI correlates with pulmonary capillary wedge pressure at rest (r = 0.48, *p* < 0.001) and during stress (r = 0.55, *p* < 0.001), validating it as a noninvasive marker of elevated filling pressures [17].

The theoretical advantage of LACI emerges from its integration of both atrial and ventricular contributions to diastolic performance [26]. While E/e’ ratio and left atrial volume index provide important information about diastolic burden, they fail to capture the dynamic interaction between chambers that LACI quantifies [17]. Importantly, LACI retains prognostic value after adjusting for conventional diastolic parameters, indicating it captures additional pathophysiological information [26]. In heart failure with preserved ejection fraction, where diastolic dysfunction represents the primary pathophysiological mechanism, LACI seems to be a promising index for early diagnosis and risk stratification [9] (Figure 1) (Supplementary Table S1).

#### 3.2.4. Dynamic Remodeling and Progressive Uncoupling

Atrioventricular uncoupling represents a progressive process characterized by maladaptive remodeling of both chambers in response to hemodynamic stress [1]. Longitudinal studies demonstrate that changes in LACI over time predict incident cardiovascular events independent of baseline values [3]. In the MESA cohort, average annualized change in LACI (ΔLACI) showed stronger associations with incident heart failure than baseline LACI alone [3]. Factors influencing the rate of uncoupling progression include underlying disease etiology, comorbidity burden, and neurohormonal activation [27]. Age and diabetes emerge as independent determinants of LACI in population studies, with accelerated uncoupling observed in older individuals and those with metabolic disease [27]. Gender differences also exist, with women demonstrating higher baseline LACI values but potentially slower progression rates [28]. Ethnicity influences both normal values and disease-related changes, with African Americans showing the highest baseline LACI and fastest progression in longitudinal follow-up [27]. Interventions targeting neurohormoral activation, including renin-angiotensin system inhibitors and mineralocorticoid receptor antagonists, may slow or reverse uncoupling, though dedicated studies examining LACI as a therapeutic endpoint remain limited [29].

### 3.3. Clinical Applications in Cardiovascular Diseases

#### 3.3.1. Heart Failure

LACI demonstrates particularly robust prognostic value in heart failure across the spectrum of ejection fraction phenotypes [5]. In patients with heart failure and left ventricular ejection fraction less than 50%, LACI independently predicts the combined endpoint of all-cause death or heart failure hospitalization with a hazard ratio of 1.77 (*p* = 0.02) after comprehensive multivariable adjustment [5]. Patients in the highest LACI tertile (>30.9%) exhibit substantially worse outcomes compared to those in lower tertiles, demonstrating the index’s ability to stratify risk within heart failure populations [5]. The median LACI of 27.1% in this cohort significantly exceeds values observed in healthy populations, reflecting the severity of atrioventricular uncoupling in established heart failure [5].

Heart failure with preserved ejection fraction represents a particularly important application for LACI, given the limitations of traditional ventricular metrics in this population [9]. In a cross-sectional study of Vietnamese patients, LACI showed exceptional diagnostic value for Heart failure with preserved Ejection Fraction (HFpEF) with an area under the curve of 0.951 and optimal threshold of 33.07% [9]. Notably, LACI values were significantly higher in HFpEF (59.16 ± 17.94%) compared to heart failure with reduced ejection fraction (41.28 ± 15.27%), reflecting the predominant role of diastolic dysfunction and atrial remodeling in preserved ejection fraction syndromes [9]. Exercise stress CMR studies demonstrate that LACI identifies HFpEF patients with high diagnostic accuracy, correlating strongly with invasive hemodynamic parameters [17]. At rest, HFpEF patients show significantly elevated LACI (45.7%) compared to those with non-cardiac dyspnea (31.6%), with values increasing further during exercise stress [17]. The ability of LACI to capture the dynamic response to physiological stress enhances its diagnostic utility in patients with exertional symptoms but normal resting hemodynamics [17] (Table 2).

Pathophysiologically, left atrial (LA) remodeling is a central driver in the advancement of heart failure (HF), especially in heart failure with preserved ejection fraction (HFpEF). In the initial phases of diastolic dysfunction, the LA makes compensatory structural and functional adjustments to maintain adequate cardiac output. However, chronic exposure to high left ventricular (LV) filling pressures ultimately leads to atrial myocardial fibrosis, decreased compliance, and heightened stiffness, which collectively trigger a pathological elevation in LACI. Over time, these maladaptive changes result in atrial dilation and the breakdown of synchronized LA-LV interactions, even in cases where LV systolic function remains intact. Because it captures both the structural alterations of the LA and the dynamic interplay between the two chambers, LACI serves as a holistic metric for tracking disease severity and customizing treatments across all HF classifications.

#### 3.3.2. Ischemic Heart Disease and Acute Myocardial Infarction

In patients following acute myocardial infarction, LACI emerges as a powerful predictor of major adverse cardiac events including death, reinfarction, and heart failure [12]. A large cohort study of 1046 AMI patients demonstrated that LACI was significantly higher in those experiencing adverse events, with an optimal cut-off of 34.7% identifying high-risk patients [12]. Greater LACI associated with MACE on univariate analysis (HR 8.1, 95% CI 3.4–14.9, *p* < 0.001) and maintained independence after adjusting for baseline confounders and left ventricular ejection fraction (HR 3.1, 95% CI 1.0–9.0, *p* = 0.049) [12]. Importantly, LACI enabled further risk stratification in high-risk patients with impaired LV systolic function (LVEF ≤ 35%), identifying a subset with particularly poor prognosis [12].

The prognostic value of both left and right atrioventricular coupling indices has been demonstrated in ST-elevation myocardial infarction [30]. In a study of 1083 AMI patients, LACI and RACI independently associated with major adverse cardiac events and provided incremental prognostic value beyond traditional risk factors [30]. The Harrell’s C index increased from 0.679 with traditional factors alone to 0.756 with addition of LACI, demonstrating significant improvement in prognostic discrimination [30]. Notably, RACI was superior to LACI in patients with right ventricular myocardial infarction (C index: 0.84 vs. 0.76), highlighting the importance of chamber-specific coupling assessment based on infarct location [30]. The relationship between LACI and clinical characteristics reveals associations with age, TIMI risk score, GRACE score, and NT-proBNP levels, suggesting integration with established risk assessment frameworks [30]. Hyperglycemia at admission exacerbates atrial dysfunction and elevates LACI, representing a modifiable risk factor that may guide therapeutic interventions [29] (Table 2).

#### 3.3.3. Cardiomyopathies (Hypertrophic, Dilated, Amyloidosis)

Hypertrophic cardiomyopathy demonstrates particularly marked atrioventricular uncoupling, with LACI serving as an independent predictor of new-onset atrial fibrillation and stroke [10]. In a CMR study of 114 HCM patients, 49% exhibited LACI greater than 40%, indicating significant uncoupling [10]. Patients with preserved coupling (LACI ≤ 40%) had substantially lower cumulative event rates compared to those with uncoupling (log-rank *p* = 0.031) [10]. After adjustment for late gadolinium enhancement, sex, and age, LACI remained independently associated with the combined endpoint of new-onset AF or stroke (HR = 23.27, *p* = 0.016) [10]. The mechanisms underlying uncoupling in HCM include left ventricular outflow tract obstruction, diastolic dysfunction from myocardial hypertrophy and fibrosis, and primary atrial myopathy [31]. Interventions such as transapical beating-heart septal myectomy improve left atrial function and reduce LACI, with preoperative LACI predicting the magnitude of strain improvement [8].

**Figure 3:**
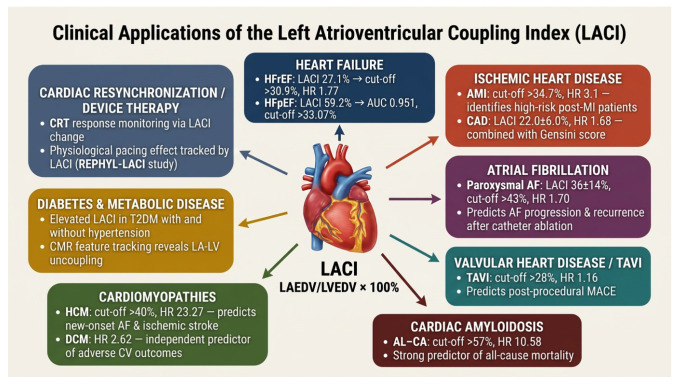
Clinical applications of LACI.

Dilated cardiomyopathy patients demonstrate elevated LACI reflecting biventricular and bi-atrial remodeling [15]. Three-dimensional echocardiographic assessment reveals that the combined atrioventricular coupling index (CACI), integrating both left and right coupling, provides a detailed prognostic discrimination [16]. In a cohort of 121 DCM patients, all three coupling indices (LACI, RACI, and CACI) independently predicted adverse events, with CACI showing the highest area under the curve (0.66) and incremental value over traditional risk factors [16]. Light-chain cardiac amyloidosis represents another cardiomyopathy where LACI demonstrates exceptional prognostic value [16]. In AL-CA patients, a median LACI of 0.57 discriminated outcomes with high accuracy, with values greater than this threshold associated with substantially increased mortality [16]. Multivariate analysis confirmed LACI as independently associated with all-cause death after adjusting for NT-proBNP, troponin T, and other clinical variables (adjusted HR: 10.58, *p* = 0.008) [16]. Furthermore, LACI enhanced risk stratification when added to traditional Mayo staging models, demonstrating incremental prognostic value [16] (Table 2 and Figure 3).

#### 3.3.4. Atrial Fibrillation and Arrhythmias

LACI serves as both a predictor of new-onset atrial fibrillation and a marker of atrial myopathy in patients with established AF [32]. The Multi-Ethnic Study of Atherosclerosis demonstrated that LACI independently predicts incident atrial fibrillation with superior discrimination compared to traditional risk scores [32]. Adjusted models incorporating LACI showed significant improvement in model discrimination compared to the CHARGE-AF score (C-statistic: 0.78 vs. 0.74) [32]. Both baseline LACI and annualized change in LACI (ΔLACI) associated strongly with AF development, with ΔLACI demonstrating a hazard ratio of 1.71 (95% CI 1.50–1.94, *p* < 0.001) [32]. These findings underscore that progressive atrioventricular uncoupling represents a critical substrate for AF initiation and maintenance [11].

In patients with established atrial fibrillation, LACI differentiates paroxysmal from persistent AF and predicts recurrence after ablation [11]. Persistent AF patients exhibit higher LACI values (0.52 ± 0.27) compared to paroxysmal AF (0.36 ± 0.14, *p* = 0.036), reflecting greater atrial and atrioventricular remodeling [11]. After radiofrequency ablation, elevated preoperative LACI independently predicts recurrence, with an optimal cut-off demonstrating good sensitivity and specificity for identifying high-risk patients [33]. Real-time three-dimensional echocardiography reveals that LACI assessed before initial ablation correlates with late recurrence, offering predictive value that guides patient selection and post-ablation surveillance [22]. Importantly, LACI captures information beyond left atrial volume alone, as it integrates both atrial enlargement and the compensatory or maladaptive changes in ventricular size that accompany AF progression [11]. The index may also guide decisions regarding rhythm versus rate control strategies, with severely elevated LACI potentially indicating advanced atrial myopathy where rhythm control is less likely to succeed [11] (Table 2 and Figure 2).

#### 3.3.5. Valvular Heart Disease and Interventional Procedures

In patients with severe aortic stenosis undergoing transcatheter aortic valve implantation (TAVI), preoperative LACI emerges as an independent risk factor for major adverse cardiac events [34]. A prospective echocardiographic study of 148 TAVI patients demonstrated that those experiencing MACE had significantly higher baseline LACI (37.84 ± 10.38) compared to event-free patients (28.18 ± 6.05, *p* < 0.001) [34]. Multivariate Cox analysis confirmed LACI as independently associated with MACE occurrence (HR: 1.16, 95% CI: 1.10–1.22, *p* < 0.001) [34]. An optimal cut-off of 28% provided acceptable sensitivity and specificity for risk stratification, with Kaplan–Meier analysis revealing significantly higher event rates in patients exceeding this threshold [34]. These findings suggest that LACI captures the burden of diastolic dysfunction and atrial remodeling that influences outcomes independent of valve hemodynamics and ventricular function [34].

The relationship between mitral regurgitation and atrioventricular coupling demonstrates the bidirectional nature of valvular-chamber interaction [35]. In type 2 diabetes patients with functional mitral regurgitation, regurgitation severity independently determines left atrial strain parameters, with moderate-to-severe MR associated with markedly impaired coupling [35]. Conversely, elevated LACI may contribute to functional MR through annular dilatation and altered ventricular geometry, creating a vicious cycle of progressive valve-chamber dysfunction [36] (Table 2).

### 3.4. Prognostic Value and Risk Stratification

#### 3.4.1. Prediction of Adverse Cardiovascular Events

Research has established LACI as an independent predictor of major cardiovascular outcomes, such as mortality, heart failure hospitalization, and atrial fibrillation recurrence. Its ability to provide incremental prognostic value over standard markers indicates that it effectively measures unique disease mechanisms like atrioventricular uncoupling [3]. Adding LACI to established risk scores could improve patient stratification—most notably in HFpEF, where current models often fail to account for the central role of the left atrium. Even in HFrEF, LACI delivers critical prognostic data that complements traditional ejection fraction measurements to better identify vulnerable subgroups [1].

In the landmark MESA study of 2250 participants free of clinical heart failure and cardiovascular disease at baseline, both LACI and change in LACI over 10 years independently predicted incident heart failure after comprehensive risk adjustment [3]. The adjusted hazard ratio for LACI was 1.44 (95% CI, *p* < 0.0001), with ΔLACI showing an even stronger association (adjusted HR 1.55, 95% CI, *p* < 0.0001) [3]. These findings established LACI as a promising predictor of subclinical cardiac dysfunction in asymptomatic individuals, enabling earlier identification of at-risk populations before clinical disease manifestation [3].

The prognostic value extends beyond heart failure to encompass atrial fibrillation, cardiovascular death, and composite endpoints [28]. In pre- and post-menopausal women from MESA, LACI independently predicted AF (HR 1.69), heart failure (HR 1.62), coronary heart disease death (HR 1.36), and hard cardiovascular disease (HR 1.30), all *p* < 0.001 [28]. Gender-specific analyses reveal that while women demonstrate higher baseline LACI values than men, the prognostic associations remain robust in both sexes [27]. In patients undergoing coronary CT angiography, LACI measured from routine acquisitions independently predicted cardiovascular death and all-cause mortality with incremental value over traditional risk factors and coronary anatomy findings [20]. A LACI threshold of 25% demonstrated optimal risk stratification, with a hazard ratio of 1.07 per 1% increment for cardiovascular death [20]. The incremental prognostic value of LACI beyond established risk assessment tools represents one of its most clinically important attributes [3]. In predicting incident heart failure, LACI-inclusive models demonstrate significant improvement in discrimination compared to traditional MESA-HF risk scores, with C-statistic increasing from 0.77 to 0.81 [3]. Similar incremental value emerges for atrial fibrillation prediction, with C-statistic improvement from 0.74 to 0.78 and NRI of 0.325 when LACI enhances the CHARGE-AF score [32].

Post-myocardial infarction risk stratification exemplifies the complementary information provided by LACI beyond ventricular function [12]. While left ventricular ejection fraction remains the cornerstone of prognostic assessment after AMI, LACI identifies high-risk patients even among those with preserved or mildly reduced LVEF [12]. In patients with LVEF > 35%, LACI still enabled significant risk stratification for major adverse cardiac events, suggesting it captures pathophysiology not reflected by systolic function alone [12]. The combination of LACI with established prognostic markers including infarct size, microvascular obstruction, and myocardial salvage provides comprehensive risk assessment integrating structural, functional, and coupling abnormalities [30].

#### 3.4.2. Comparison with Isolated Atrial and Ventricular Parameters

LAVI is a well-established marker of chronic diastolic burden and atrial remodeling. However, LAVI only reflects the structural adaptation of a single chamber. Prognostic models incorporating LACI demonstrated better discrimination and risk reclassification compared to models relying on LA volume index or LV end-diastolic volume alone, reflecting LACI’s unique ability to capture maladaptive chamber interaction rather than isolated structural remodeling. The superiority of LACI over isolated chamber measurements emerges from multiple comparative studies [3]. In the MESA cohort, the prognostic value of LACI exceeded that of individual LA or LV parameters for predicting incident heart failure [3]. Models incorporating LACI demonstrated better discrimination and reclassification compared to models using LA volume index or LV end-diastolic volume alone [3]. This superior performance reflects LACI’s ability to capture the interaction between chambers rather than isolated structural remodeling [12]. In patients following acute myocardial infarction, LACI provided incremental value beyond left ventricular ejection fraction, demonstrating that coupling indices capture distinct pathophysiological information not reflected by systolic function [12].

Left atrial reservoir strain, a widely studied marker of atrial function, demonstrates complementary but distinct prognostic value compared to LACI [37]. While LA reservoir strain primarily reflects atrial compliance and contractility, LACI integrates both atrial remodeling and ventricular adaptation, providing a more comprehensive assessment of chamber interaction [26]. In heart failure with preserved ejection fraction, LA reservoir strain shows the highest diagnostic accuracy among individual parameters, yet the LASr/LVGLS ratio (a coupling metric) provides superior prognostic value with a C-index of 0.670 [37].

Left Ventricular Ejection Fraction (LVEF) remains the clinical cornerstone for evaluating systolic function and guiding prognostic assessment. Nevertheless, LVEF frequently fails to reflect early atrioventricular uncoupling or isolated diastolic dysfunction. In patients following acute myocardial infarction, LACI provided significant incremental prognostic value beyond LVEF, demonstrating that coupling indices capture distinct pathophysiological information entirely missed by systolic metrics. Importantly, LACI successfully identifies high-risk patients even among cohorts with preserved or only mildly reduced LVEF (>35%), enabling further risk stratification for major adverse cardiac events where standard ejection fraction metrics offer a false sense of security. Direct comparisons between LACI and left ventricular global longitudinal strain reveal that both parameters independently predict adverse events, but LACI was observed to demonstrate stronger associations in populations with predominantly diastolic dysfunction [5]. In a cohort of 478 heart failure patients with LVEF < 50%, LACI remained significantly associated with outcomes after adjusting for LA reservoir strain, LVEF, and other established predictors (HR 1.77, *p* = 0.02) [5]. Notably, LACI offers practical advantages including simpler measurement, independence from strain software platforms, and derivability from standard imaging protocols without specialized post-processing [13].

#### 3.4.3. Incremental Predictive Value

LACI emerged from large population-based studies demonstrating that the ratio of atrial to ventricular volumes yields prognostic insights extending far beyond traditional risk factors. Its capacity to enhance established risk stratification models is consistently evident across a broad spectrum of cardiovascular conditions. For instance, incorporating LACI into the MESA-HF score for incident heart failure significantly improves prognostic discrimination, increasing the C-statistic from 0.77 to 0.81 [38]. Similarly, it refines the prediction of incident atrial fibrillation by elevating the established CHARGE-AF C-statistic from 0.74 to 0.78, yielding a Net Reclassification Improvement of 0.325 [32]. This robust incremental predictive value translates equally well to ischemic and structural heart diseases; integrating LACI with traditional risk factors increases the Harrell’s C-index from 0.679 to 0.756 in patients with ST-elevation myocardial infarction, while a LACI threshold of 25% significantly improves mortality risk models over standard coronary anatomy findings in patients undergoing routine coronary CT angiography [12]. Furthermore, LACI enhances traditional Mayo staging for patients with light-chain cardiac amyloidosis [16].

#### 3.4.4. Special Populations (Chronic Kidney Disease, Diabetes, Hypertension)

Chronic kidney disease patients demonstrate particularly elevated LACI values reflecting the compounded effects of volume overload, uremic cardiomyopathy, and accelerated cardiovascular remodeling [39]. In CKD stage 4–5 patients, those with concomitant diabetes show notably higher LACI compared to CKD patients without diabetes, suggesting additive deleterious effects on atrioventricular coupling [39]. During 21-month follow-up, LACI emerged as an independent predictor of major adverse cardiac events in this high-risk population [39]. The prognostic value of LACI appears particularly robust in CKD patients with heart failure and preserved ejection fraction, where traditional markers often fail to adequately stratify risk [39]. In this population, LACI ≥ 0.235 demonstrated discriminative capability for moderate-to-severe diastolic dysfunction (AUC = 0.739), while an optimal cut-off of 0.26 effectively identified patients at elevated risk for adverse cardiovascular events [36].

Diabetes mellitus independently associates with impaired atrioventricular coupling, with hyperglycemia exacerbating LA dysfunction and elevating LACI even in the absence of overt cardiac disease [40]. Speckle tracking echocardiography studies reveal that diabetic patients exhibit greater LA and LV stiffness along with increased LACI compared to healthy controls [40]. The combination of diabetes and hypertension demonstrates synergistic effects on coupling, with DM + HP patients showing significantly decreased LA strain and increased LACI compared to diabetic patients without hypertension [41]. Hypertension independently contributes to decreased LA booster strain and increased LACI in diabetic individuals, indicating potential atrioventricular coupling alterations [41,42]. These findings suggest that LACI may serve as an early marker of cardiac involvement in metabolic disease, potentially guiding intensification of preventive therapies before development of symptomatic heart failure [39,40].

### 3.5. Future Directions and Clinical Translation

#### 3.5.1. Standardization of Measurement Protocols and Reference Values

Widespread clinical implementation of LACI requires establishment of standardized measurement protocols and normative reference values across diverse populations [1,43]. Current variability in imaging techniques, chamber segmentation algorithms, and post-processing software create challenges for comparing results between studies and establishing universal diagnostic thresholds [9,13,43]. Collaborative efforts involving major cardiovascular imaging societies should prioritize development of consensus guidelines for LACI acquisition and analysis [1]. These protocols must address technical considerations including optimal imaging planes, temporal resolution requirements, and standardized approaches to atrial and ventricular segmentation [13]. Validation of modality-specific reference values and prognostic thresholds represents another critical need [43]. While studies consistently demonstrate prognostic value of LACI, optimal cut-offs vary considerably between populations and imaging modalities [1]. Development of cross-modality conversion algorithms could enable translation of findings between echocardiography, CMR, and CT, facilitating broader clinical application [13,44]. Finally, cost-effectiveness analyses comparing LACI-guided management strategies to conventional approaches would provide important data supporting clinical adoption and reimbursement decisions [13].

LACI cutoff values differ significantly between CT, CMR, and echocardiography primarily due to systematic differences in how each modality measures the left atrial and left ventricular volumes used to calculate the index. Two-dimensional transthoracic echocardiography (2D TTE) systematically underestimates left atrial volumes compared to CMR and CT. In a meta-analysis of 17 studies with 1203 patients, TTE underestimated LA volume by −20 mL (95% CI −30, −11) and indexed LA volume by −9 mL/m^2^ (95% CI −13, −5) compared to CMR (*p* < 0.001) [45]. This led to misclassification of LA enlargement in 38% cases [46]. In patients with mitral regurgitation, the mean difference was even larger: TTE showed mean LAVI 47.1 ± 20.8 mL/m^2^ while CMR showed 70 ± 20.3 mL/m^2^ (*p* < 0.001), with a mean difference of approximately 20 mL/m^2^ [47]. Similarly, compared to CT, TTE underestimated LA volume by −23.7 mL (95% CI: −64.9 to 17.5, *p* < 0.0001) [46]. In patients with permanent atrial fibrillation, TTE underestimated maximum LA volume (60 vs. 73 mL/m^2^ for CMR and 60 vs. 80 mL/m^2^ for CT, *p* < 0.0001) [48].

2D echocardiography relies on geometric assumptions (e.g., biplane area-length method) that presume symmetric LA morphology. However, the left atrium has complex three-dimensional anatomy with non-uniform remodeling, making one- or two-dimensional measurements inherently inaccurate [49]. CMR and CT offer superior spatial resolution and true three-dimensional imaging, allowing more accurate delineation of LA borders. CMR is considered the gold standard for volumetric assessment due to its high spatial resolution [45,50]. A critical source of discrepancy is how each modality handles endocardial trabeculae. CMR’s high spatial resolution allows trabeculae to be included within the ventricular cavity, while echocardiography’s limited resolution causes trabeculae to be incorporated into the myocardial tracing, systematically reducing measured cavity volumes [51,52]. Similar systematic differences exist for LV volume measurements. Echocardiography underestimates LV end-diastolic volume by approximately 9.4 mL and end-systolic volume by 3.5 mL compared to CMR [53]. Even 3D echocardiography, which is more accurate than 2D, still underestimates LV volumes by approximately 14–39 mL for end-diastolic volume depending on the analysis method used [54,55]. Because LACI is calculated as the ratio of LA to LV end-diastolic volumes, the differential underestimation of these volumes by echocardiography affects the resulting LACI value. Since both the numerator (LA volume) and denominator (LV volume) are underestimated, but potentially by different proportions, the ratio changes.

#### 3.5.2. Integration with Artificial Intelligence and Machine Learning

Artificial intelligence and machine learning technologies offer transformative potential for advancing LACI assessment and clinical application [19]. AI-enhanced echocardiography platforms enable automated chamber segmentation and volume quantification, reducing measurement variability and analysis time while improving reproducibility [19]. These systems can calculate LACI in real-time during image acquisition, facilitating point-of-care risk assessment and clinical decision-making [19]. Machine learning algorithms trained on large datasets can identify optimal combinations of LACI with other imaging and clinical variables, creating integrated risk prediction models with superior performance compared to traditional approaches [19].

Deep learning applications extend beyond measurement automation to enable prediction of future coupling deterioration and cardiovascular events [19]. Neural networks can analyze temporal sequences of imaging studies to identify patterns of progressive uncoupling associated with adverse outcomes [2]. Integration of LACI with multi-omic data including genomics, proteomics, and metabolomics may enable precision phenotyping of atrioventricular uncoupling mechanisms [1]. Such comprehensive approaches could identify patients most likely to benefit from specific therapeutic interventions targeting coupling improvement [2].

#### 3.5.3. Therapeutic Implications and Treatment Monitoring

LACI holds promise as both a therapeutic target and a marker for monitoring treatment response [1,38]. Interventions that reduce left ventricular filling pressures or improve diastolic function may reverse atrioventricular uncoupling, with LACI serving as a sensitive indicator of therapeutic efficacy [56]. In hypertensive patients treated with spironolactone, improvements in LA function and atrioventricular coupling occurred independent of blood pressure reduction, suggesting direct myocardial effects of mineralocorticoid receptor antagonism [38,56]. Similar principles may apply to other neurohormonal antagonists including ACE inhibitors, angiotensin receptor blockers, and SGLT2 inhibitors [2]. Prospective trials examining whether LACI-guided therapy improves outcomes compared to conventional management would provide definitive evidence supporting therapeutic targeting of coupling [1,3].

Device-based therapies including cardiac resynchronization therapy demonstrate complex effects on LACI that require further investigation [24]. While CRT promotes favorable ventricular remodeling and improves clinical outcomes, some studies report paradoxical LACI worsening despite symptom improvement [23]. Understanding whether this represents true deterioration in coupling versus differential chamber remodeling rates will inform optimal patient selection and programming strategies [24]. In patients undergoing structural interventions such as TAVI or mitral valve repair, serial LACI assessment could identify those achieving optimal hemodynamic benefit versus persistent chamber uncoupling requiring additional therapy [34].

#### 3.5.4. Integration into Patient-Centered Care and Current Clinical Practice

LACI enables identification of subclinical atrioventricular uncoupling, allowing preemptive intervention before symptomatic HF develops. Serial LACI measurements guide therapy titration, enhancing personalized treatment plans and shared decision-making. As a result, by integrating a mechanistic understanding of LA-LV coupling, clinicians can predict patient-specific responses to interventions, tailor medications, or schedule follow-ups, emphasizing individualized outcomes. Finally, echocardiography-based LACI facilitates routine monitoring without invasive procedures, aligning with patient safety and preference principles. Given all the data analyzed in the manuscript LACI has gained increasing attention in contemporary clinical cardiology due to its ability to simultaneously evaluate atrial and ventricular interactions. Current clinical applications of LACI extend across a spectrum of cardiovascular conditions, including heart failure, atrial fibrillation, hypertrophic cardiomyopathy, and post-myocardial infarction remodeling. Elevated LACI values correlate with adverse outcomes, serving as an independent prognostic marker for hospitalization, major adverse cardiovascular events, and mortality. Moreover, LACI has demonstrated utility in monitoring treatment responses, such as improvements in atrioventricular coupling following cardiac resynchronization therapy. Its measurement is feasible through multimodal imaging platforms, including echocardiography, cardiac magnetic resonance, and computed tomography, making it both accessible for routine clinical assessment and valuable for risk stratification, early diagnosis, and the guidance of personalized therapeutic strategies. While this review focuses on the imaging and hemodynamic evaluation of atrioventricular coupling, it is important to acknowledge that pharmacological interventions are also evolving to target the molecular underpinnings of cardiac remodeling. Recent studies have highlighted the role of SGLT2 inhibitors and other novel agents in modulating pathways such as oxidative stress, apoptosis, and inflammation [57,58]. Future research should explore how these molecular-level improvements translate into macroscopic functional changes in LACI, potentially establishing the index as a sensitive tool for monitoring pharmacological efficacy.

### 3.6. Limitations of LACI

The Left Atrioventricular Coupling Index (LACI) provides insight into how the left atrium and ventricle work together, but several factors limit its routine clinical application. Measurements are highly dependent on imaging technique and operator skill, with echocardiography relying on geometric assumptions, CMR and CT being accurate but costly or less accessible, and arrhythmias introducing variability. LACI values are also influenced by heart rate, blood volume, and other comorbid conditions such as mitral valve disease or atrial fibrillation, making interpretation context-specific.

Additionally, there is no universally accepted reference range, and different formulas are used across studies, which complicates standardization. Most existing prognostic evidence was derived mostly from observational research with different cut-off values used for different cardiac pathologies (such as heart failure with reduced and preserved ejection fraction or hypertrophic cardiomyopathy) based on the observer’s experience and familiarization with this index or the cardiac pathology solely, and prospective validation is limited. Therefore, while LACI can enhance understanding of cardiac function, it should be interpreted alongside other echocardiographic parameters and clinical data to guide patient management effectively. LACI is influenced by numerous cardiovascular conditions and comorbidities including obesity, hypertension, diastolic dysfunction and loading conditions. This makes it challenging to determine whether an abnormal LACI reflects intrinsic left atrial dysfunction, left ventricular pathology, or systemic factors. Most validation studies have included only patients in sinus rhythm, significantly limiting the applicability of LACI in patients with atrial fibrillation—a population where left atrial assessment is particularly important. Moreover, to become a clinical tool widely available, randomized trials should be held to establish a universally accepted reference range of normal values and standardized measurement protocols.

## 4. Conclusions

The left atrioventricular coupling index represents a significant advance in cardiovascular assessment, providing a simple yet powerful metric that captures the essential interaction between the left atrium and ventricle [1]. By quantifying the ratio of atrial to ventricular end-diastolic volumes, LACI reflects the integrated nature of cardiac performance beyond single-chamber evaluation [2,3,42]. Evidence presented in our review, derived mostly from observational trials, given that LACI is not a part of the European Society of Cardiology/AHA guidelines yet, demonstrates its promising nature as a prognostic value of early diagnosis of atrioventricular uncoupling across the spectrum of cardiovascular disease. Yet, given the absence of standardized measurement protocols, large, randomized trials are needed to further validate this index and define standardized reference values, establishing it as a standard addition to the current guidelines of indexes used for assessment of atrioventricular function. Moreover, most clinicians and imaging specialists should be further familiarized with is application to become a standard addition to the current clinical practice.

## Figures and Tables

**Table 1:** Presentation of all available imaging modalities for assessment of LACI.

Imaging Modality	Advantages	Limitations	Typical LACI Values	Best Clinical Context
2D Echocardiography	Widely available, no radiation, real-time, low cost	Geometric assumptions, image quality dependent, operator variability	17.0 ± 8.0% (normal)	Screening, serial monitoring, bedside assessment
3D Echocardiography	Direct volumetric assessment, no geometric assumptions, good correlation with CMR	Limited availability, requires expertise, image quality dependent	16.5 ± 7.5% (normal)	Comprehensive functional assessment, research studies
Cardiac MRI	Gold standard accuracy, tissue characterization, excellent reproducibility	High cost, contraindications, limited availability, time-consuming	13.8–18.0% (normal)	Definitive assessment, tissue characterization, research
Cardiac CT	Integrated coronary assessment, good volumetric accuracy, widely available	Radiation exposure, contrast required, limited temporal resolution	15.1 ± 6.4% (normal)	Combined anatomic-functional assessment, contraindication to MRI

**Table 2:** Applications of LACI in clinical practice.

Disease Category	Condition	Typical LACI (%)	Prognostic Cut-Off (%)	Hazard Ratio (HR)	Clinical Implications
Heart Failure	HFrEF	27.1 (19.9–34.5)	>30.9	1.77	Risk stratification for all-cause mortality and heart failure hospitalization
	HFpEF	59.2 ± 17.9	>33.07	—	High diagnostic accuracy for HFpEF (AUC = 0.951)
Ischemic Heart Disease	Acute Myocardial Infarction (AMI)	Variable	>34.7	3.1 (adjusted)	Identifies high-risk patients following myocardial infarction
	Coronary Artery Disease (CAD)	22.0 ± 6.0	Disease-specific	1.68	Improves risk stratification when combined with Gensini score
Cardiomyopathies	Hypertrophic Cardiomyopathy (HCM)	Variable	>40	23.27	Predicts new-onset atrial fibrillation and ischemic stroke
	Dilated Cardiomyopathy (DCM)	Elevated	—	2.62	Independent predictor of adverse cardiovascular outcomes
	Light-chain Cardiac Amyloidosis (AL-CA)	Variable	>57	10.58	Strong predictor of all-cause mortality
Atrial Fibrillation	Paroxysmal AF	36 ± 14	>43	1.70	Predicts AF progression and recurrence after catheter ablation
	Persistent AF	52 ± 27	—	—	Marker of advanced atrial myopathy
Valvular Heart Disease	Transcatheter Aortic Valve Implantation (TAVI)	Variable	>28	1.16	Predicts post-procedural major adverse cardiovascular events (MACE)

## Data Availability

The data presented in this study are available in the article and its Supplementary Materials.

## Supplementary Materials

The following supporting information can be downloaded at: https://www.mdpi.com/article/10.3390/life16050722/s1, Table S1: Comparison of Echocardiographic and Imaging Parameters for the Assessment of Diastolic Function [59,60,61].

## References

[1] Afana A., Hudelo J., Gonc T., Garot Ô., Soulat G., Fauvel C., Dacher J.-N., Coisne A., Pontana F., Bohbot Y. (2025). Left and right atrioventricular coupling: State-of-the-art review. Eur. Heart J. Cardiovasc. Imaging.

[2] Liu X., Wang J., Tong Y., Wang S. (2025). The power of the left atrioventricular coupling index in cardiovascular disease. Front. Cardiovasc. Med..

[3] Pezel T., Ambale Venkatesh B., Kato Y., De Vasconcellos H.D., Heckbert S.R., Wu C.O., Post W.S., Bluemke D.A., Cohen-Solal A., Henry P. (2021). Left Atrioventricular Coupling Index to Predict Incident Heart Failure: The Multi-Ethnic Study of Atherosclerosis. Front. Cardiovasc. Med..

[4] Qin S., Zhang L., Ji M., Wu Z., Lin Y., He Q., Xie M., Li Y. (2025). Clinical Utility of Atrioventricular Coupling Index in Cardiovascular Disease. J. Am. Heart Assoc..

[5] Kasa G., Teis A., De Raffele M., Cediel G., Juncà G., Lupón J., Santiago-Vacas E., Codina P., Bayés-Genis A., Delgado V. (2025). Prognostic value of left atrioventricular coupling index in heart failure. Eur. Heart J. Cardiovasc. Imaging.

[6] Zakeri R., Moulay G., Chai Q., Ogut O., Hussain S., Takahama H., Lu T., Wang X.L., Linke W.A., Lee H.C. (2016). Left Atrial Remodeling and Atrioventricular Coupling in a Canine Model of Early Heart Failure with Preserved Ejection Fraction. Circ. Heart Fail..

[7] Kato Y., Ambale-Venkatesh B., Noda C., Ortman J., Thomas T., Chamera E., Kassai Y., Liu C.-Y., Lima J.A.C. (2025). Abstract 4364017: Left atrial passive strain rate by cardiac magnetic resonance imaging better reflects left ventricular diastolic function than left atrial reservoir strain: A cross-sectional study in a post-COVID-19 cohort. Circulation.

[8] Zhao Y., Xiang J.Y., Pan Z., Li C., Huang L., Tang D., Luo Y., Xiang C., Zhou X., Wei X. (2025). Impact of transapical beating-heart septal myectomy on left atrial remodeling and atrioventricular coupling in hypertrophic obstructive cardiomyopathy. J. Cardiovasc. Magn. Reson..

[9] Nguyen Ngoc Dang H., Viet Luong T., Khanh Tran H., Ha Tuyet Le N., Hoang Nhat Nguyen M., Chi Doan T., Minh Nguyen H. (2025). Left atrioventricular coupling index measured by echocardiography in heart failure with preserved ejection fraction. Sci. Rep..

[10] De Raffele M., Teis A., Weerts J., Niro L., Kasa G., Junca G., Cediel G., Bertini M., Bayes-Genis A., Delgado V. (2024). Left atrioventricular coupling index is an independent associate of new-onset AF and stroke in hypertrophic cardiomyopathy: A CMR study. Eur. Heart J..

[11] Karanikola A.E., Tsiachris D., Sakalidis A., Kordalis A., Tzortzi M., Antoniou C.K., Laina A., Argyriou N., Botis M., Doundoulakis I. (2025). Increased left atrioventricular coupling index as a marker of atrial myopathy in patients with atrial fibrillation. Eur. Heart J..

[12] Lange T., Backhaus S.J., Schulz A., Evertz R., Kowallick J.T., Bigalke B., Hasenfuß G., Thiele H., Stiermaier T., Eitel I. (2023). Cardiovascular magnetic resonance-derived left atrioventricular coupling index and major adverse cardiac events in patients following acute myocardial infarction. J. Cardiovasc. Magn. Reson..

[13] Poręba M., Kraik K., Zasoński I., Ratajczyk O., Paździerz Ł., Chachaj A., Poręba R., Gać P. (2025). The Possibilities and Importance of Assessing the Left Atrioventricular Coupling Index Using Various Diagnostic Imaging Methods in an Adult Population: A Comprehensive Review. J. Cardiovasc. Dev. Dis..

[14] Anwar A.M., Alshammakh M.S., Eyaz M., Al-Katheri A., Abdelfattah A.N., Ali M.A.M., Albakri I. (2025). Normal reference values of left atrioventricular coupling index on two-Dimensional echocardiography. Int. J. Cardiovasc. Imaging.

[15] Vîjîiac A., Scărlătescu A.I., Petre I.G., Vîjîiac C., Vătășescu R.G. (2024). Three-Dimensional Combined Atrioventricular Coupling Index-A Novel Prognostic Marker in Dilated Cardiomyopathy. Biomedicines.

[16] Meng F., Li J., Zhao R., Wu Y., Liu Y., Yang Y., Yang Y., Zhou N., Dong L., Kong D. (2025). Left atrioventricular coupling index assessed with three-dimensional echocardiography: A prognostic marker of short-term outcomes in light-chain cardiac amyloidosis. Amyloid.

[17] Backhaus S.J., Lange T., Schulz A., Evertz R., Frey S.M., Hasenfuß G., Schuster A. (2023). Cardiovascular magnetic resonance rest and exercise-stress left atrioventricular coupling index to detect diastolic dysfunction. Am. J. Physiol. Heart Circ. Physiol..

[18] Lange T., Backhaus S.J., Schulz A., Hashemi D., Evertz R., Kowallick J.T., Hasenfuß G., Kelle S., Schuster A. (2024). CMR-based cardiac phenotyping in different forms of heart failure. Int. J. Cardiovasc. Imaging.

[19] Qin S., Li D., Feng Q., Zhang Y., Zhou D., Liu S. (2025). Artificial intelligence-enhanced three-dimensional echocardiography reveals left atrial-ventricular coupling index as a novel prognostic marker in coronary artery disease. BMC Cardiovasc. Disord..

[20] Pezel T., Dillinger J.G., Toupin S., Mirailles R., Logeart D., Cohen-Solal A., Unger A., Canuti E.S., Beauvais F., Lafont A. (2023). Left atrioventricular coupling index assessed using cardiac CT as a prognostic marker of cardiovascular death. Diagn. Interv. Imaging.

[21] Cheładze P., Fułek M., Fułek K., Poręba R., Gać P. (2025). Association Between Cardiovascular Risk Assessed by the SCORE System and Cardiac Computed Tomography-Derived Left Atrioventricular Coupling Index. Diagnostics.

[22] Zhu R.Y., Hu H.T., Zhang A.Y., Shen D., Hu W.S., Li X.Y., Sun H., Zhou C. (2025). Predictive Value of Left Atrial Coupling Index Applied to Real-Time Three-Dimensional Echocardiography for Late Recurrence After Ablation in Patients with Paroxysmal Atrial Fibrillation. Echocardiography.

[23] Sanna G., Raccis M., Oliva V., Rossi S., Dossi F., Maggi R., Donateo P., Parodi G. (2024). Effects of cardiac resynchronization therapy and physiological pacing on left atrioventricular coupling index: The REPHYL-LACI study. Eur. Heart J..

[24] Chiu C.S.L., Gerrits W., Wouters P.C., Cramer M.J., Van Der Harst P., Vernooy K., Van Stipdonk A.M.W., Van Halm V.P., Van Dijk V., Ghani A. (2025). Left atrioventricular coupling index in cardiac resynchronization therapy responders and non-responders. EP Eur..

[25] Zheng H., Zhang L., Wu X., Zheng X. (2025). Echocardiography derived-left atrial stiffness index as a more cost-effective and powerful approach compared to brain natriuretic peptide for predicting left atrioventricular uncoupling in patients with acute ischemic stroke. Quant. Imaging Med. Surg..

[26] Fortuni F., Biagioli P., Myagmardorj R., Mengoni A., Lupi A., Chua A.P., Zuchi C., Sforna S., Di Pietro I., Viti C. (2024). Left atrioventricular coupling index: Association with diastolic dysfunction and prognostic implications in heart failure patients. Eur. Heart J..

[27] Pezel T., Venkatesh B.A., Vasconcellos H.D., Kato Y., Post W.S., Wu C.O., Heckbert S.R., Bluemke D.A., Cohen-Solal A., Logeart D. (2022). Determinants of left atrioventricular coupling index: The Multi-Ethnic Study of Atherosclerosis (MESA). Arch. Cardiovasc. Dis..

[28] Pezel T., Michos E.D., Varadarajan V., Shabani M., Venkatesh B.A., Vaidya D., Kato Y., De Vasconcellos H.D., Heckbert S.R., Wu C.O. (2022). Prognostic value of a left atrioventricular coupling index in pre- and post-menopausal women from the Multi-Ethnic Study of Atherosclerosis. Front. Cardiovasc. Med..

[29] Han P.L., Li K., Jiang Y., Jiang L., Tang X., Guo Y.K., Li Y., Yang Z.G. (2025). Left atrioventricular coupling and left atrial abnormality in patients with acute myocardial infarction with and without hyperglycemia assessed with 3.0 T cardiac magnetic resonance imaging feature tracking. Quant. Imaging Med. Surg..

[30] Wu J.P., Zhao Y., Gu X.Y., Chen B.H., Wu R., Andong A.L., Shi R.Y., Wu C.W., Dai Y.S., Zhao L. (2026). Atrioventricular Coupling Index: A Novel Approach to Risk-Stratification for Major Adverse Cardiovascular Events in Patients with ST-Elevation Myocardial Infarction. J. Magn. Reson. Imaging.

[31] Tran T.V., Djaileb L., Riou L., Lantuejoul L.R., Giai J., Barone-Rochette G. (2024). Coronary microvascular dysfunction as assessed by multimodal diagnostic imaging in patients with hypertrophic cardiomyopathy is related to the severity of cardiac dysfunction. Microcirculation.

[32] Pezel T., Ambale-Venkatesh B., Quinaglia T., Heckbert S.R., Kato Y., de Vasconcellos H.D., Wu C.O., Post W.S., Henry P., Bluemke D.A. (2022). Change in Left Atrioventricular Coupling Index to Predict Incident Atrial Fibrillation: The Multi-Ethnic Study of Atherosclerosis (MESA). Radiology.

[33] Li A., Zhang M., Ning B. (2024). Predictive value of the left atrioventricular coupling index for recurrence after radiofrequency ablation of paroxysmal atrial fibrillation. J. Cardiothorac. Surg..

[34] Wu B., Huang T., Zeng D., Fang Q., Cai Y., Chang S., Li Y., Luo H., Huang L., Chen M. (2025). Prognostic value of left atrioventricular coupling index in patients undergoing transcatheter aortic valve implantation: A prospective echocardiographic study. Quant. Imaging Med. Surg..

[35] Zhang Y., Li X.M., Shen M.T., Huang S., Li Y., Yang Z.G. (2022). Atrioventricular coupling and left atrial abnormality in type 2 diabetes mellitus with functional mitral regurgitation patients verified by cardiac magnetic resonance imaging. Cardiovasc. Diabetol..

[36] Wang Y.H., Dong Y., Li G.Y., Ma C.Y. (2025). Unveiling the Left Atrioventricular Coupling Index: A Promising Marker for Diastolic Dysfunction and Prognosis. J. Am. Soc. Echocardiogr..

[37] Liu M., Lin J., Cai Y., He J., Lu M., Zhao Y., Tao J., Yang W., Zhu Z., Wang Y. (2025). Atrioventricular and Ventricular Mechanical Interdependence Assessment by Automated Function Imaging in Heart Failure with Preserved Ejection Fraction. Echocardiography.

[38] Pezel T., Venkatesh B.A., De Vasconcellos H.D., Kato Y., Shabani M., Xie E., Heckbert S.R., Post W.S., Shea S.J., Allen N.B. (2021). Left Atrioventricular Coupling Index as a Prognostic Marker of Cardiovascular Events: The MESA Study. Hypertension.

[39] Gao X., Xie A., Xiao W., Ji L., Li H., Zou A., Miao Z., Zhang X., Yang S., Yu S. (2025). A novel index evaluating left atrioventricular coupling function in chronic kidney disease with diabetes patients. Sci. Rep..

[40] Dang H.N.N., Luong T.V., Ho B.A. (2024). Evaluation of the relationship between left atrial stiffness, left ventricular stiffness, and left atrioventricular coupling index in type 2 diabetes patients: A speckle tracking echocardiography study. Front. Cardiovasc. Med..

[41] Shi R., Jiang Y.N., Qian W.L., Guo Y.K., Gao Y., Shen L.T., Jiang L., Li X.M., Yang Z.G., Li Y. (2023). Assessment of left atrioventricular coupling and left atrial function impairment in diabetes with and without hypertension using CMR feature tracking. Cardiovasc. Diabetol..

[42] Zornitzki L., Topilsky Y. (2024). Left Atrioventricular Coupling Index: When Minimal Left Atrial Volume Is Actually ‘More’ Than Maximal Left Atrial Volume. J. Am. Soc. Echocardiogr..

[43] Dong T.X., Li S.W., Pan X.F., Wang C.F., Liu Y., Wu J., Guan X.P., Zhang S.L., Zuo P.F., Liu Y.L. (2025). Normal Values of Echocardiographic Left Atrioventricular Coupling Index and Left Atrial Stiffness Index Reflecting Left Ventricular Diastolic Function: A Multicenter Study. J. Am. Soc. Echocardiogr..

[44] Fortuni F., Bernetti C., Carluccio E. (2025). The emerging role of left atrioventricular coupling index in heart failure: A new frontier for cardiac magnetic resonance. Eur. Heart J. Cardiovasc. Imaging.

[45] Mahmod M., Bull S., Kailayanathan T., Davis T.A., Borlotti A., Popescu I.A., Das I., Wamil M., Brears H.T., Banerjee R. (2025). Left atrial volume quantification by transthoracic echocardiography versus cardiovascular magnetic resonance: A systematic review and meta-analysis. Int. J. Cardiovasc. Imaging.

[46] Arsanjani R., Flint N., Beigel R., Khachatryan T., Shalev A., Shturman A., Lee C., Rader F., Berman D.S., Heo R. (2019). Comparison of Accuracy of Left Atrial Area and Volume by Two-dimensional Trans-thoracic Echocardiography Versus Computed Tomography. Am. J. Cardiol..

[47] El Mathari S., Hopman L.H.G.A., Bouchnaf C., Heidendael J.F., Nederveen A.J., van Ooij P., Selder J.L., van Loon R.B., Götte M.J.W., Kluin J. (2024). Clinical implications of different methods to assess left atrial remodeling: A comparative study between echocardiography and cardiac magnetic resonance imaging for left atrial volume index quantification. Int. J. Cardiol..

[48] Agner B.F.R., Kühl J.T., Linde J.J., Kofoed K.F., Åkeson P., Rasmussen B.V., Jensen G.B., Dixen U. (2014). Assessment of left atrial volume and function in patients with permanent atrial fibrillation: Comparison of cardiac magnetic resonance imaging, 320-slice multi-detector computed tomography, and transthoracic echocardiography. Eur. Heart J. Cardiovasc. Imaging.

[49] Goette A., Kalman J.M., Aguinaga L., Akar J., Cabrera J.A., Chen S.A., Chugh S.S., Corradi D., D’aVila A., Dobrev D. (2017). EHRA/HRS/APHRS/SOLAECE expert consensus on atrial cardiomyopathies: Definition, characterization, and clinical implication. Heart Rhythm..

[50] Bax J.J., Di Carli M., Narula J., Delgado V. (2019). Multimodality imaging in ischaemic heart failure. Lancet.

[51] Polte C.L., Lagerstrand K.M., Gao S.A., Lamm C.R., Bech-Hanssen O. (2015). Quantification of Left Ventricular Linear, Areal and Volumetric Dimensions: A Phantom and in Vivo Comparison of 2-D and Real-Time 3-D Echocardiography with Cardiovascular Magnetic Resonance. Ultrasound Med. Biol..

[52] Marwick T.H. (2018). Ejection Fraction Pros and Cons: JACC State-of-the-Art Review. J. Am. Coll. Cardiol..

[53] Guo F.Q., Wu B.L., Liu X.W., Pan T., Gao B.L., Li C.Y. (2023). Three-Tesla magnetic resonance imaging of left ventricular volume and function in comparison with computed tomography and echocardiography. Medicine.

[54] Argulian E., Narula J. (2021). Advanced Cardiovascular Imaging in Clinical Heart Failure. JACC Heart Fail..

[55] Dorosz J.L., Lezotte D.C., Weitzenkamp D.A., Allen L.A., Salcedo E.E. (2012). Performance of 3-dimensional echocardiography in measuring left ventricular volumes and ejection fraction: A systematic review and meta-analysis. J. Am. Coll. Cardiol..

[56] Girard A.A., Denney T.S., Gupta H., Dell’Italia L.J., Calhoun D.A., Oparil S., Sharifov O.F., Lloyd S.G. (2024). Spironolactone improves left atrial function and atrioventricular coupling in patients with resistant hypertension. Int. J. Cardiovasc. Imaging.

[57] Deng J., Liu Q., Ye L., Wang S., Song Z., Zhu M., Qiang F., Zhou Y., Guo Z., Zhang W. (2024). The Janus face of mitophagy in myocardial ischemia/reperfusion injury and recovery. Biomed. Pharmacother..

[58] Wang Z., Liu J., Chen Y., Tang Y., Chen T., Zhou C., Wang S., Chang R., Chen Z., Yang W. (2025). From physiology to pathology: Emerging roles of GPER in cardiovascular disease. Pharmacol. Ther..

[59] Thomas L., Marwick T.H., Popescu B.A., Donal E., Badano L.P. (2019). Left Atrial Structure and Function, and Left Ventricular Diastolic Dysfunction: JACC State-of-the-Art Review. J. Am. Coll. Cardiol..

[60] Nagueh S.F., Sanborn D.Y., Oh J.K., Anderson B., Billick K., Derumeaux G., Klein A., Koulogiannis K., Mitchell C., Shah A. (2025). Recommendations for the Evaluation of Left Ventricular Diastolic Function by Echocardiography and for Heart Failure with Preserved Ejection Fraction Diagnosis: An Update From the American Society of Echocardiography. J. Am. Soc. Echocardiogr..

[61] Redfield M.M., Borlaug B.A. (2023). Heart Failure with Preserved Ejection Fraction: A Review. JAMA.

