# Supplementary material for: Left Atrioventricular Coupling Index: A Comprehensive Review of the Literature
Source: Life (Basel). 2026 Apr 24;16(5):722. doi: 10.3390/life16050722 (PMC13208728; doi:10.3390/life16050722)
Supplement: Supplementary file 1 [file life-16-00722-s001.zip › life-4211672-supplementary.pdf]

Supplementary Table S1: Comparison of Echocardiographic and Imaging Parameters for the Assessment of Diastolic Function.

Parameter	What It Measures	Advantages	Limitations	Prognostic Value	Normal Values/Cutoffs	References
**LACI** (Left Atrioventricular Coupling Index)	LA-to-LV volume ratio; reflects LA/LV coupling and atrial Frank-Starling mechanism	• Integrates both LA and LV remodeling<br>• Only volumetric parameter differentiating HFpEF from non-cardiac dyspnea at rest<br>• Correlates with PCWP (r = 0.48–0.55)<br>• Amenable to stress testing<br>• Independent predictor after adjusting for clinical factors	• Multiple definitions exist (LAmin/LVEDV vs LAVi/a′)<br>• Lack of standardized reference values until recently<br>• Limited validation in arrhythmias<br>• Modality-specific cutoffs required (echo vs CMR vs CT)	• Independent predictor of CV hospitalization/death in HFpEF<br>• Stratifies risk across terciles in stress CMR<br>• Predicts outcomes in CKD + HFpEF (HR 1.32 per quartile)	• Echo: Mean 16.4 ± 7.3% (healthy); >33% for HFpEF (AUC 0.951)<br>• CMR: >25–26% abnormal<br>• Increases with age; higher in women	[43]
**LAVI** (Left Atrial Volume Index)	LA structural remodeling; chronic elevation of LA pressure	• Excellent feasibility and reproducibility<br>• Superior to M-mode LA dimension<br>• Widely used in clinical trials<br>• Predicts severe LVDD (AUC 0.97–0.98)<br>• Established reference values	• Reflects "legacy" of past elevated pressures, not current LAP<br>• Slow/incomplete reverse remodeling<br>• Increased in non-cardiac conditions (AF, bradycardia, high-output states, MV disease, athletes)<br>• Weak correlation with LAP changes<br>• Normal in early grade I LVDD	• Independent predictor of all-cause mortality<br>• Predicts CV death/HF hospitalization in HFpEF trials<br>• Less discriminative than LA strain or stiffness indices	• Normal: 34 mL/m²<br>• Mild: 34–41 mL/m²<br>• Moderate: 42–48 mL/m²<br>• Severe: >48 mL/m²<br>• Surgical threshold (MR): >60 mL/m²	[59]
**E/e′ ratio**	Estimates LV filling pressure (LAP)	• Widely available<br>• Part of standard diastolic assessment<br>• Validated against invasive PCWP<br>• Independent predictor of outcomes in HFpEF	• Load-dependent<br>• Affected by heart rate, rhythm, BP<br>• Average E/e′ >14 needed for elevated LAP<br>• Gray zone (8–14) common<br>• Exercise echo has low sensitivity (34%) for HFpEF diagnosis	• Independent predictor of HF hospitalization/CV death in HFpEF<br>• Inferior to LA stiffness index (E/e′/PALS) for prognosis	• Normal: 8<br>• Indeterminate: 8–14<br>• Elevated LAP: >14<br>• Diastolic dysfunction: E/e′ ≥ 8	[60]
**TR velocity (TRV)/PASP**	Estimates pulmonary artery systolic pressure; reflects elevated LAP	• Direct measure of RV systolic pressure<br>• Part of ASE algorithm for LAP estimation<br>• TRV ≥ 2.8 m/s supports elevated LAP	• Requires adequate TR jet<br>• May need contrast enhancement<br>• Affected by pulmonary vascular disease<br>• Not obtainable in all patients<br>• Blooming artifacts can cause errors	• Independent predictor of HF hospitalization/CV death<br>• PASP ≥ 35 mmHg associated with worse outcomes<br>• Inferior to LA stiffness index for prognosis	• Normal PASP: 35 mmHg<br>• Elevated LAP: PASP ≥ 35 mmHg or TRV ≥ 2.8 m/s	[61]
**e′ velocity** (mitral annular)	LV relaxation, restoring forces, lengthening load	• Direct measure of LV relaxation<br>• First step in ASE diastolic algorithm<br>• Widely available<br>• Less load-dependent than mitral inflow	• Affected by multiple factors beyond relaxation Age-dependent<br>• Regional variation (septal vs lateral)<br>• Can be normal in early LVDD	• Reduced e′ associated with worse outcomes<br>• Part of composite diastolic assessment	• Septal e′ 7 cm/s abnormal<br>• Lateral e′ 10 cm/s abnormal<br>• Average e′ 9 cm/s abnormal	[60]
